# Supplementary figures and images for: Diacylglycerol Acyltransferase 3(DGAT3) Is Responsible for the Biosynthesis of Unsaturated Fatty Acids in Vegetative Organs of Paeonia rockii
Source: Int J Mol Sci. 2022 Nov 19;23(22):14390. doi: 10.3390/ijms232214390 (PMC9692848; doi:10.3390/ijms232214390)

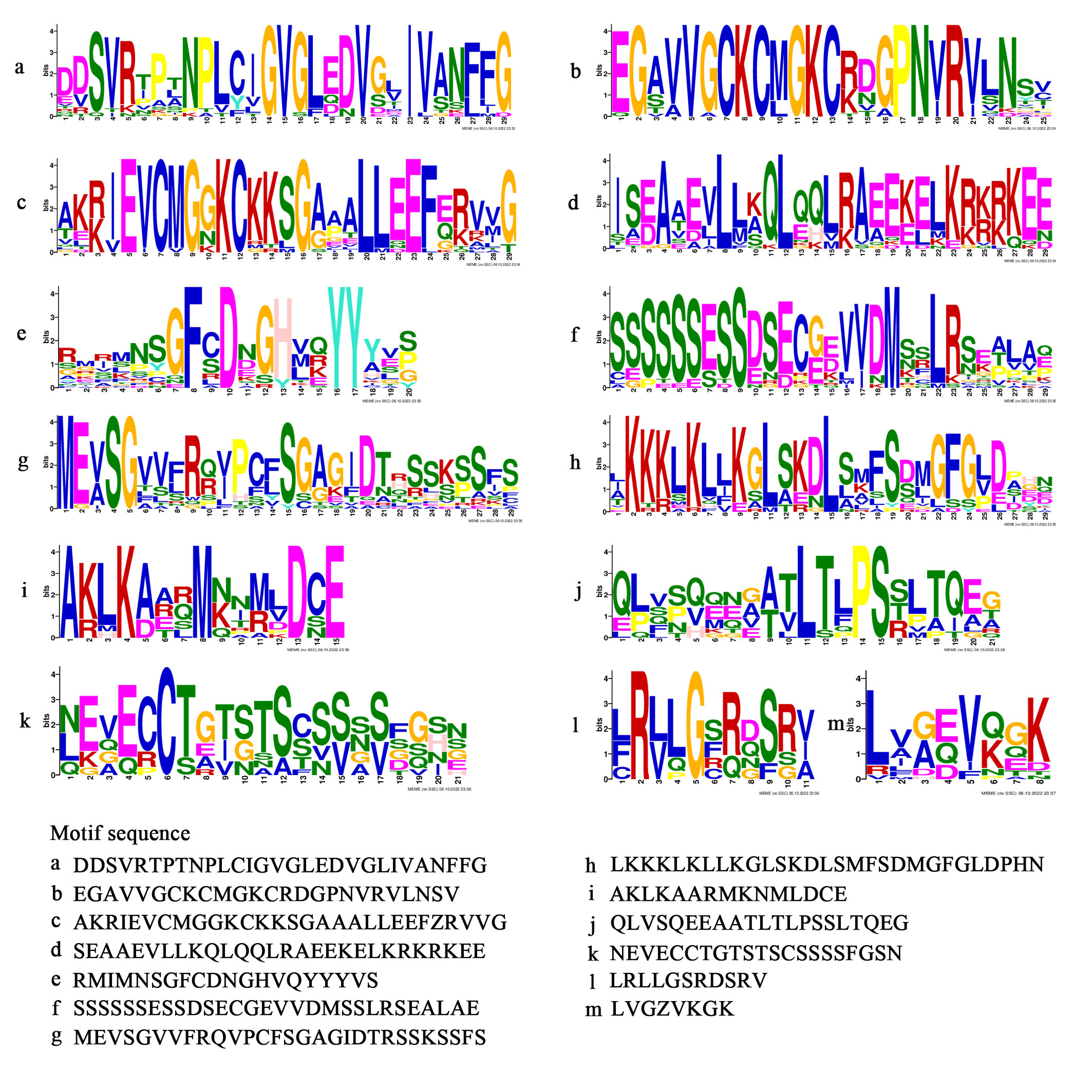

Supplement: Supplementary file 1 [file ijms-23-14390-s001.zip › Figure S1.jpg]

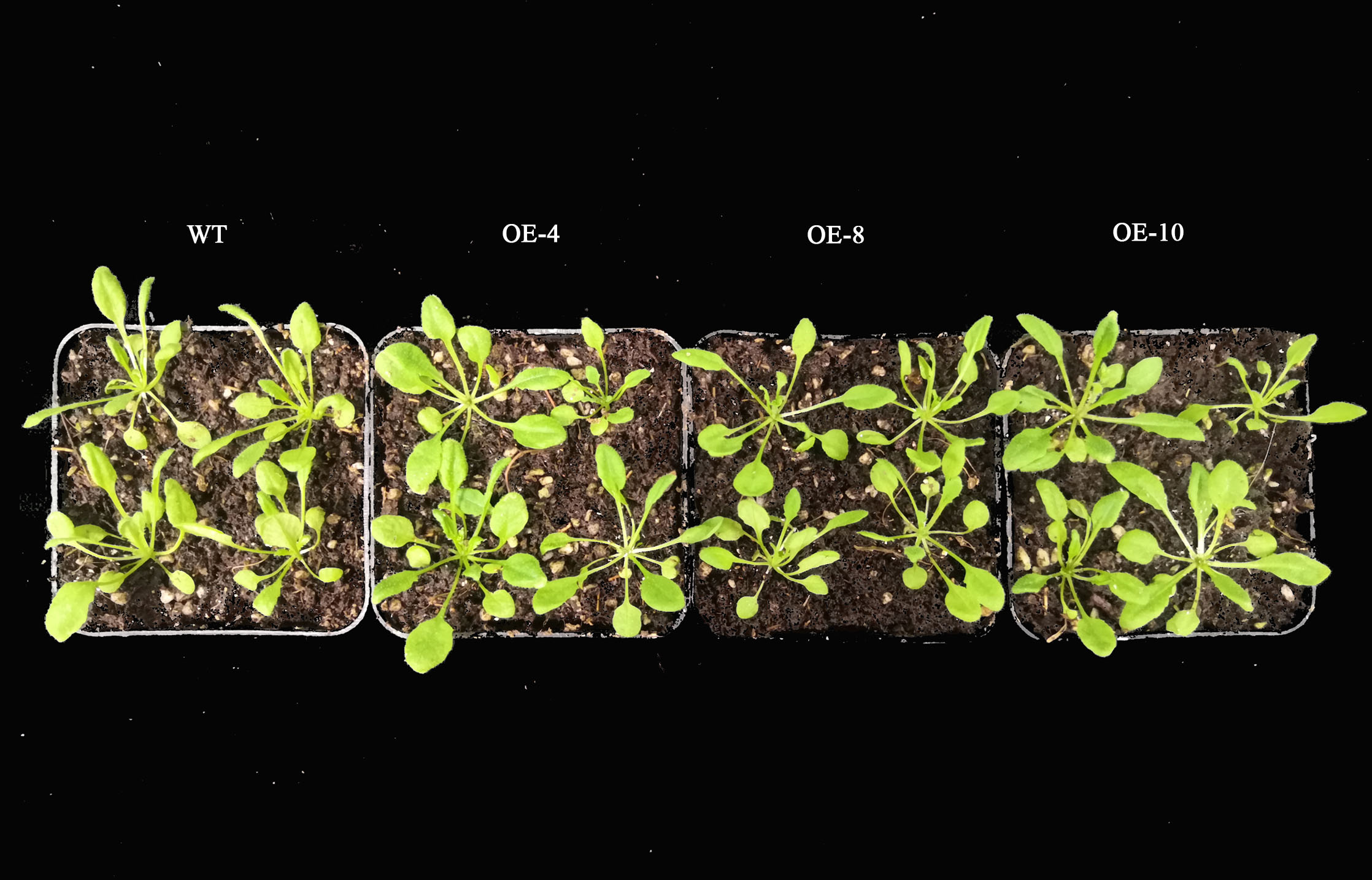

Supplement: Supplementary file 1 [file ijms-23-14390-s001.zip › Figure S2.jpg]

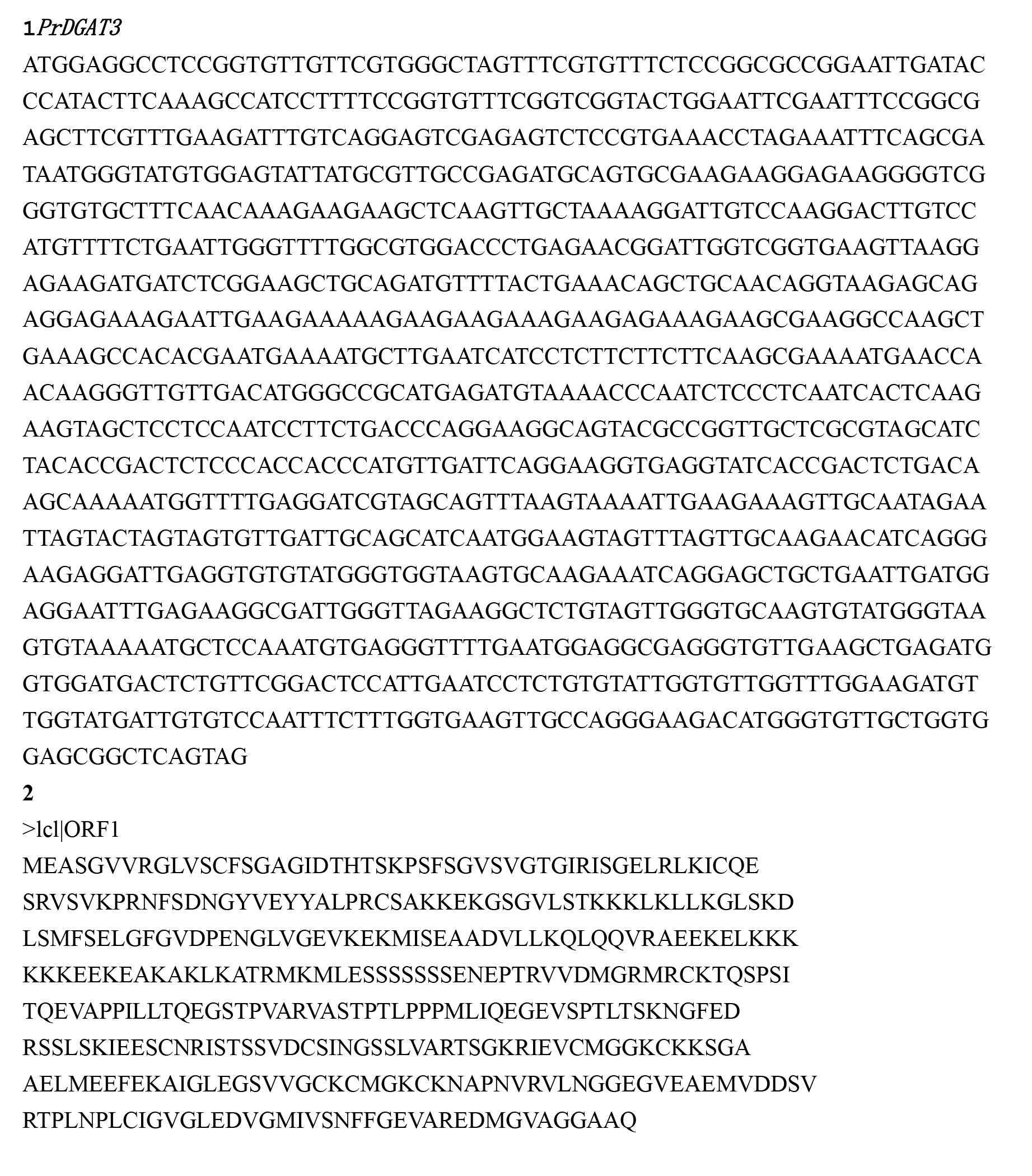

Supplement: Supplementary file 1 [file ijms-23-14390-s001.zip › Figure S4.jpg]
